# The Food Matrix Protects Dark Chocolate Flavan-3-Ols and Onion Flavonols from Degradation During In Vitro Gastrointestinal Digestion

**DOI:** 10.3390/biology15010088

**Published:** 2025-12-31

**Authors:** Alice Cattivelli, Melissa Zannini, Maddalena De Angeli, Roberta Trovato, Angela Conte, Davide Tagliazucchi

**Affiliations:** Nutritional Biochemistry Lab, Department of Life Sciences, University of Modena and Reggio Emilia, Via Amendola 2, 42122 Reggio Emilia, Italy; alice.cattivelli@unimore.it (A.C.); melissa.zannini@unimore.it (M.Z.); maddalena.deangeli@unimore.it (M.D.A.); roberta.trovato.tr@gmail.com (R.T.); angela.conte@unimore.it (A.C.)

**Keywords:** mass spectrometry, phenolic compounds, food matrix, procyanidins, quercetins

## Abstract

The bioaccessibility of flavonoids (namely, their release from the food matrix and their stability under gastrointestinal conditions) is pivotal in establishing their bioavailability and biological effects. Bioaccessibility is affected by several factors, including the food matrix. Previous studies suggested that flavonols and flavan-3-ols were higher in solid foods rather than in beverages. Therefore, this study investigated the bioaccessibility of red-skinned onion flavonols and dark chocolate flavan-3-ols during in vitro gastrointestinal digestion of whole foods and the corresponding phenolic compounds extracts, with the aim of ascertaining a possible food matrix effect. Results showed that the presence of the food matrix protected flavonols and flavan-3-ols from degradation during digestion. The bioaccessibility of total flavonols and flavan-3-ols determined by mass spectrometry was 79.0% and 80.8% for red-skinned onion and dark chocolate, respectively, whereas it was 57.5% and 47.3% for the corresponding extracts. Degradation of flavonols occurred mainly during intestinal digestion by deglycosylation and oxidative reactions, whereas for flavan-3-ols, it occurred during gastric digestion mainly by hydrolysis. Therefore, this study highlighted the importance of the food matrix in protecting flavonols and flavan-3-ols from degradation during digestion, underscoring the significance of consuming phenolic compounds in whole foods rather than supplements or extracts.

## Simple Summary

Flavonoids are health-promoting compounds found in vegetables that possess numerous biological activities and are able to prevent the onset of several chronic degenerative diseases, such as cancer and cardiovascular diseases. Red-skinned onion and dark chocolate are two widely consumed foods particularly rich in flavonols and flavan-3-ols, respectively. To exert their biological activities, these compounds have to be released from the food matrix and stable to the gastrointestinal conditions (i.e., have to be bioaccessible). The release and stability of flavonoids are dependent on several factors, including the food matrix. This study examined how much of these compounds can be released and remain stable during lab-simulated digestion of the whole foods and their extracts, to understand if the food matrix affected this process. The bioaccessibility of flavonols and flavan-3-ols was 79.0% in red-skinned onion and 80.8% in dark chocolate after digestion. In comparison, lower values were observed for their phenolic extracts, with bioaccessibility of 57.5% for onion and 47.3% for chocolate extracts. Most of the flavonols in the extracts degraded in the intestines following deglycosylation and oxidative degradation, while most of the flavan-3-ols in the stomach were degraded mainly by hydrolysis reaction. Results showed that the food matrix helped protect flavonols and flavan-3-ols from degradation during digestion.

## 1. Introduction

Phenolic compounds are plant secondary metabolites widely distributed throughout the plant kingdom and are daily consumed by humans through the intake of vegetables and derived foods and beverages [1]. Beyond their ecological importance and their role in protecting plants against pathogens, phenolic compounds have received significant attention in recent years due to their potential protective effects against the onset of chronic degenerative diseases in humans [1]. Numerous in vitro and in vivo studies have attributed various health-promoting effects to phenolic compounds, including the protection against cardiovascular disease onset and cancer development and progression [2,3,4,5,6].

Among the different phenolic compound classes, the estimated higher dietary intake was observed for flavonoids and phenolic acids [7,8]. Within flavonoids, flavan-3-ols and flavonols were the subclasses that contributed most significantly to dietary intake [9,10]. The major contributors to flavonol intake were leafy vegetables, onions, tea, and apples, whereas for flavan-3-ols, the main contributors were tea, chocolate, and cocoa-based products [5,10,11]. Recent studies have linked higher flavonol intake with reduced risks of all-cause, cancer-specific, and cardiovascular-disease-specific mortality, a lower incidence of type 2 diabetes and obesity, and improvement of cognitive abilities [12,13,14,15,16,17]. Similarly, flavan-3-ols intake has been associated with decreased all-cause mortality, lower cancer incidence, and reduced risk of type 2 diabetes and cardiovascular diseases [5,18,19,20].

A critical issue influencing the biological effects of phenolic compounds is their bioavailability. Bioavailability refers to the fraction of a consumed compound that is absorbed in the gastrointestinal tract and becomes available to exert its biological activity in the body [1]. For absorption in the gastrointestinal tract, a compound must be released from the food matrix (i.e., made bioaccessible) and then cross the intestinal barrier. Bioaccessibility is defined as the amount of phenolic compounds released from the solid matrix in the gut, making them available for absorption [21]. Consequently, bioavailability represents the proportion of bioaccessible phenolic compounds that are absorbed into the bloodstream and can be distributed and metabolized within the body [22]. Therefore, bioaccessibility is of paramount importance in determining the bioavailability of phenolic compounds and their ability to exert health benefits at the gut level [23]. The bioaccessibility of phenolic compounds depends on several factors, including the structure of the compound, the chemical–physical conditions in the gastrointestinal tract, and the food matrix [24,25].

In particular, the bioaccessibility of phenolic compounds is intricately linked to the food matrix, as they may interact with macromolecules, such as proteins, fibers, and lipids [26]. The effect is strictly related to the macromolecules considered. In general, proteins reduced the bioaccessibility of phenolic compounds, whereas lipids increased their bioaccessibility and bioavailability [27]. In the case of proteins, the reduced bioaccessibility of phenolic compounds has been attributed to the formation of complexes between phenolic compounds and proteins, which lead to the formation of insoluble aggregates [26,27]. Conversely, interactions between lipids and phenolic compounds may promote micellization and increase their solubility, thereby protecting them from degradation during gastrointestinal transit [26,27]. Meanwhile, fibers exhibited varying effects on the bioaccessibility of phenolic compounds, with increases or decreases observed depending on the specific fiber type examined [27]. Water-soluble fibers may form soluble complexes with phenolic compounds, increasing their solubility and protecting them from degradation during digestion [26,27]. In contrast, when phenolic compounds are incorporated in insoluble fibers, their solubility and bioaccessibility decrease [26,27]. In addition, the digestibility of plant-based foods is highly influenced by their structure, which must be broken down and modified by mastication and digestive enzymes to release phenolic compounds [28,29]. The chemical structure of phenolic compounds also plays a critical role in bioaccessibility. For example, the presence of a catechol group in the structure of phenolic compounds (as often occurs in the B-ring of flavonols and flavan-3-ols) can promote oxidative reactions during digestion that degrade the compounds, decreasing their bioaccessibility [23,30]. Moreover, additional reactions such as hydrolysis, deacetylation, demethylation, and deglycosylation may occur during in vitro digestion of flavonoids [31]. In this context, the food matrix could protect phenolic compounds from degradation, for example, by binding them to macromolecules and releasing them slowly during digestion. Furthermore, previous studies have shown that some phenolic compounds (including flavonols and flavan-3-ols) exhibited higher bioaccessibility when present in a solid food matrix rather than in a beverage or an extract, suggesting a possible protective effect of the food matrix against their degradation [23,25].

Therefore, the aim of this study was to compare the bioaccessibility of individual phenolic compounds when digested within a solid food matrix versus a liquid medium after their extraction from the solid food. The selected foods were dark chocolate as a source of flavan-3-ols and red-skinned onion as a source of flavonols. In addition to being particularly rich in flavonoids, these matrices were selected because previous studies have demonstrated a high bioaccessibility of flavonols and flavan-3-ols during the in vitro digestion of these foods [23,32].

## 2. Materials and Methods

### 2.1. Materials and Sample Preparation

Enzymes and chemicals for in vitro gastrointestinal digestion were purchased from Sigma (Milan, Italy). Solvents for extraction and mass spectrometry analysis were obtained from BioRad (Hercules, CA, USA). Dark chocolate (74%) and red-skinned onion were purchased at a local supermarket (Reggio Emilia, Italy). Red-skinned onion (Tropea cultivar) was peeled, washed, and dried on absorbent paper, whereas dark chocolate was used as such. All samples were stored at −80 °C until use.

### 2.2. Preparation of the Phenolic Compounds Chemical Extract from Red-Skinned Onion and Dark Chocolate

The extraction of phenolic compounds from red-skinned onion was performed as reported in Cattivelli et al. using a methanol/water/formic acid (70:28:2, *v*/*v*) extraction solution [33]. Samples (15 g) were mixed with 30 mL of extract solution and homogenized with an Ultra-Turrax at 6000 rpm. Next, the mixtures were incubated under stirring at 37 °C for 30 min and centrifuged for 20 min at 4 °C and 6000 *g*. Phenolic compounds from chocolate were extracted as reported in Martini et al. [34]. Firstly, dark chocolate (10 g) was melted at 50 °C for 10 min before adding 20 mL of the extract solution. After 30 min of incubation at 37 °C under stirring, the mixture was centrifuged under the same conditions reported above. Supernatants from the extracts were subsequently evaporated using a rotary evaporator and resuspended in an amount of water equal to the original weight of the sample (i.e., 15 mL of water for red-skinned onion and 10 mL of water for dark chocolate). In this way, 1 mL of resuspended extract corresponded to 1 g of the original sample (i.e., red-skinned onion and dark chocolate).

### 2.3. In Vitro Gastro-Intestinal Digestion

In vitro gastrointestinal digestion was conducted following the INFOGEST 2.0 protocol [35]. Food samples and resuspended extracts (1 g or 1 mL) were mixed with 1 mL of simulated salivary fluid, homogenized with a pestle, and incubated for 2 min at 37 °C in a rotating wheel (10 rpm) after the addition of 150 U/mL of salivary α-amylase. Dark chocolate (1 g) was melted at 50 °C for 10 min, then 1 mL of simulated salivary fluid was added. Gastric digestion was performed by adding 2 mL of simulated gastric fluid to the bolus. After that, the pH was corrected to 3 with HCl 6 mol/L, and 2000 U/mL of pepsin was added. The bolus was then incubated for 120 min at 37 °C in a rotating wheel (10 rpm). Intestinal digestion was performed by adding 4 mL of simulated intestinal fluid, bringing the pH to 7.5 with concentrated NaOH, and adding pancreatin at 200 U/mL based on trypsin activity. The chyme was then incubated for 120 min at 37 °C in a rotating wheel (10 rpm). Digested samples were stored at –80 °C for high-resolution mass spectrometry analysis of phenolic compounds and spectrophotometric assays. Digestions were carried out in triplicate for each sample.

### 2.4. Total Phenolic Compounds Quantification and Antioxidant Activity Assays

#### 2.4.1. Quantification of Total Phenolic Compounds

Total phenolic compounds in the extracts and digested samples were quantified by the Folin–Ciocalteu assay as previously described [36]. The results were expressed as mg of gallic acid equivalent per 100 g of food.

#### 2.4.2. Determination of the Radical Scavenging Activity by ABTS Assay

The radical scavenging ability of the extracts and digested samples was determined using the ABTS assay, as described in Re et al. [37]. The ABTS radical scavenging ability was reported as mg of Trolox equivalent per 100 g of food.

#### 2.4.3. Assessment of Fe^3+^ Reducing Ability

The reducing ability of the extracts and digested samples was determined using the ferric-reducing antioxidant power (FRAP) assay, according to Benzie and Strain [38]. Data were reported as mg of FeSO_4_ equivalent per 100 g of food.

### 2.5. High-Resolution Mass Spectrometry Analysis of Flavan-3-Ols and Flavonols

Phenolic compounds were identified and quantified through high-resolution mass spectrometry analysis as described by Martini et al. and Cattivelli et al. [23,39]. Results were expressed as μmol/100 g of food or extract. The bioaccessibility index (BI) was calculated as reported by Cattivelli et al. [38]. Quantification was performed by building external calibration curves with the available standard compounds as reported in Supplementary Table S1. The standard used for quantification and the mass spectrometry data are reported in Supplementary Table S1.

### 2.6. Statistics

Quantitative data about phenolic compounds and colonic metabolites are presented as mean ± SD. ANOVA (Univariate analysis of variance) followed by Tukey’s post hoc test was carried out via GraphPad Prism 10 (GraphPad Software, San Diego, CA, USA), and the differences were considered significant when *p* < 0.05. The Shapiro–Wilk test for normality was applied before the ANOVA.

## 3. Results and Discussion

### 3.1. Effect of In Vitro Gastrointestinal Digestion on Total Phenolic Compounds and Antioxidant Activity in Red-Skinned Onion and Red-Skinned Onion Phenolic Compounds Extract

**Figure 1:**
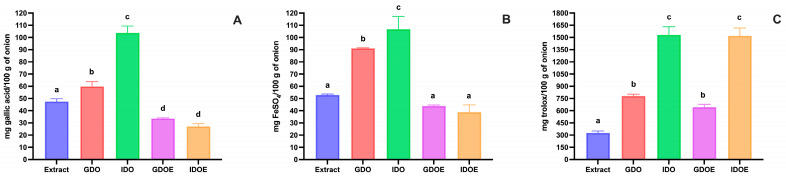
Effect of in vitro gastrointestinal digestion on the changes in total phenolic compounds and antioxidant activity in red-skinned onion and red-skinned onion phenolic compounds extract. (**A**) Total phenolic compounds determined by the Folin–Ciocalteau assay. (**B**) Antioxidant activity determined by the FRAP assay. (**C**) Antioxidant activity determined by the ABTS assay. Extract: onion phenolic compounds extract; GDO: gastric digested onion; IDO: intestinal digested onion; GDOE: gastric digested onion extract; IDOE: intestinal digested onion extract. Error bars, mean ± S.D. Different letters indicated statistically significant differences (*p* < 0.05). Significant differences were determined by ANOVA (Univariate analysis of variance) followed by Tukey’s post hoc test.

Chemical extraction of phenolic compounds from red-skinned onion resulted in a total phenolic compounds value of 47.78 ± 2.33 mg of gallic acid equivalent/100 g of onion (Figure 1A). A wide variation in the total phenolic content of red-skinned onion has been reported in the literature, with values ranging from 10 to 65 mg/100 g [40,41,42]. This great variability may be due to differences in the tested cultivars and agro-environmental factors.

Following in vitro digestion of red-skinned onion, a significantly higher amount (*p* < 0.05) of total phenolic content was observed after in vitro gastric digestion, compared to the extract, with a further significant increase (*p* < 0.05) of about 1.7 times with respect to the gastric digestion, observed at the end of intestinal digestion (Figure 1A). This increase can be due to the release from onion of Folin-reactive substances that were not present in the chemical extract or to the hydrolysis of onion proteins by the digestive enzyme that released shorter peptides and/or amino acids that can react with the Folin–Ciocalteau reagent.

A different behavior was observed when the red-skinned onion phenolic compounds extract was in vitro digested (Figure 1A). A significant decrease (*p* < 0.05) of about 30% in the total phenolic content was observed after gastric digestion, with respect to the extract. The total amount of phenolic compounds further decreased, although not significantly, at the end of intestinal digestion, resulting in a loss of about 43% compared to the undigested extract. These data suggest that some phenolic compounds in the red-skinned onion phenolic compounds extract may be degraded during in vitro digestion, indicating a potential preventive effect of the food matrix.

Antioxidant activity data, obtained by the FRAP assay, followed the same trend as the total phenolic content, as reported in Figure 1B. Whereas some differences were found in the ABTS assay for antioxidant activity. In this case, also for the in vitro digested red-skinned onion phenolic compounds extract, a significant increase (*p* < 0.05) in radical scavenging activity was observed after in vitro gastric and intestinal digestion (Figure 1C). This may indicate that potential degradation products of phenolic compounds during the digestion of the extract may have higher radical scavenging activity than the original compounds.

### 3.2. Effect of In Vitro Gastrointestinal Digestion on Total Phenolic Compounds and Antioxidant Activity in Dark Chocolate and Dark Chocolate Phenolic Compounds Extract

**Figure 2:**
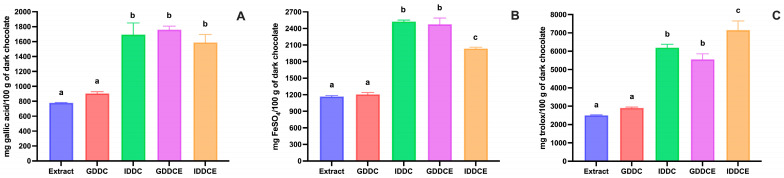
Effect of in vitro gastrointestinal digestion on the changes in total phenolic compounds and antioxidant activity in dark chocolate and dark chocolate phenolic compounds extract. (**A**) Total phenolic compounds determined by the Folin–Ciocalteau assay. (**B**) Antioxidant activity determined by the FRAP assay. (**C**) Antioxidant activity determined by the ABTS assay. Extract: dark chocolate phenolic compounds extract; GDDC: gastric digested dark chocolate; IDDC: intestinal digested dark chocolate; GDDCE: gastric digested dark chocolate extract; IDDCE: intestinal digested dark chocolate extract. Error bars, mean ± S.D. Different letters indicated statistically significant differences (*p* < 0.05). Significant differences were determined by ANOVA (Univariate analysis of variance) followed by Tukey’s post hoc test.

As reported in Figure 2A, the total amount of phenolic compounds in dark chocolate determined after chemical extraction was 779.57 ± 2.97 mg of gallic acid equivalent/100 g of dark chocolate. A wide range in total phenolic content of dark chocolate was observed, depending on cocoa percentage, processing method, and extraction method [32,43,44].

When dark chocolate was in vitro digested, a small, not significant increase in total phenolic content was observed after gastric digestion (~16% increase), whereas, after intestinal digestion, the total phenolic compounds value almost doubled compared to gastric digestion (Figure 2A). In contrast, following in vitro digestion of dark chocolate phenolic compounds extract, a 2.3-fold increase in total phenolic content was observed already after gastric digestion, and the value remained constant after intestinal digestion (Figure 2A). This increase in total phenolic content may be due to the hydrolysis of dark chocolate melanoidins that may release both phenolic compounds and low-molecular-weight Maillard reaction products that reacted with the Folin–Ciocalteau reagent [45,46]. The observed differences between dark chocolate and its phenolic compounds extract may result from the gradual release and intestinal hydrolysis of melanoidins from the solid food matrix, whereas these compounds were already present in a solubilized form and were hydrolyzed earlier, during gastric digestion. No significant differences (*p* > 0.05) in total phenolic content were observed between dark chocolate and dark chocolate phenolic compounds extract at the end of the digestion (Figure 2A).

Furthermore, the antioxidant activity data obtained with the ABTS and FRAP assays followed the same trend as the total phenolic content data during in vitro digestion of both dark chocolate and its phenolic compounds extract (Figure 2B,C).

### 3.3. Identification and In Vitro Bioaccessibility of Individual Flavonols in Red-Skinned Onion and Red-Skinned Onion Phenolic Compounds Extract

A total of 16 flavonols were identified in the red-skinned onion phenolic compounds extract (Table 1). The total amount of flavonols identified by mass spectrometry was 44.03 ± 1.41 μmol/100 g of onion (Table 1). The most representative compounds were quercetin-3-O-glucoside-4′-O-glucoside and quercetin-4′-O-glucoside, which accounted for 44.3% and 40.6% of total flavonols, respectively (Table 1). Furthermore, an appreciable amount of isorhamnetin-4′-O-hexoside was detected, accounting for 5.7% of total flavonols (Table 1). The flavonols profile of the red-skinned onion phenolic compounds extract was consistent with previously reported data [23,33,47].

**Figure 3:**
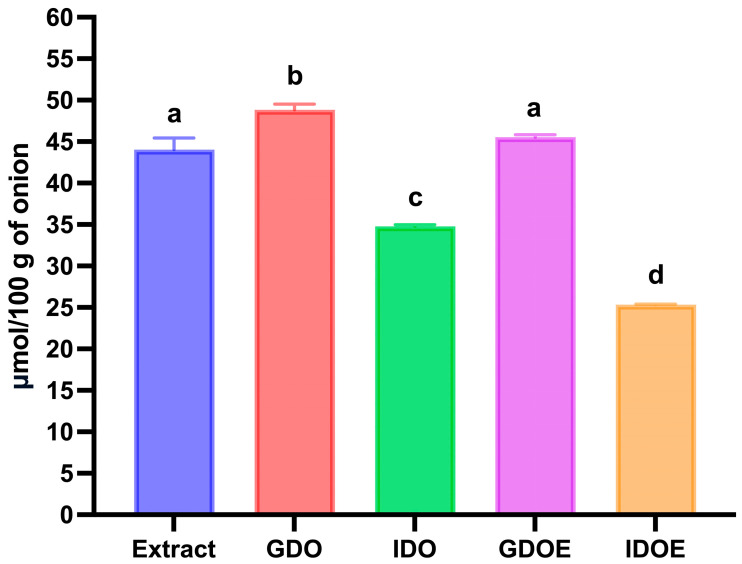
Effect of in vitro gastrointestinal digestion on the changes in total phenolic compounds determined by mass spectrometry in red-skinned onion and red-skinned onion phenolic compounds extract. Extract: onion phenolic compounds extract; GDO: gastric digested onion; IDO: intestinal digested onion; GDOE: gastric digested onion extract; IDOE: intestinal digested onion extract. Error bars, mean ± S.D. Different letters indicated statistically significant differences (*p* < 0.05). Significant differences were determined by ANOVA (Univariate analysis of variance) followed by Tukey’s post hoc test.

After in vitro gastric digestion of red-skinned onion, a bioaccessibility index slightly above 100% was observed for total flavonols, indicating that these compounds were fully released and remained stable during this phase of digestion (Table 1 and Figure 3). Regarding the two major flavonols, quercetin-3-O-glucoside-4′-O-glucoside was detected in significantly lower amounts after gastric digestion than in the extract (*p* < 0.05; bioaccessibility index of 86.5%), whereas quercetin-4′-O-glucoside showed a significant increase (*p* < 0.05; bioaccessibility index of 116.2%). (Table 1). Furthermore, a significant increase (*p* < 0.05) in the concentration of quercetin-3-O-glucoside (bioaccessibility index of 170.4%) and quercetin aglycone (bioaccessibility index of 215.4%) was recorded after in vitro gastric digestion. These results suggest the likely occurrence of hydrolysis reactions at the expense of quercetin-mono-hexosides and quercetin-di-hexosides (Table 1). In particular, an almost net molar conversion of quercetin di-hexosides (2.64 μmol/100 g decrease relative to the extract) to quercetin mono-hexosides (≈3 μmol/100 g increase relative to the extract) was observed. Specifically, quercetin-3-O-hexoside-4′-O-hexoside was deglycosylated predominantly to quercetin-4′-O-hexoside, with only a minor formation of quercetin-3-O-hexoside (Table 2). As reported by Rohn et al. [48], the hexoside in the 4′-*O*-position exhibits greater resistance to deglycosylation than that in the 3-O-position. Deglycosylation of quercetin-hexosides during in vitro gastrointestinal digestion has been previously documented in onion, apple, and pure compounds [40,49,50]. Previous studies demonstrated that hydrolysis of quercetin-hexosides took place under the acidic conditions of the gastric milieu [50,51]. Similarly, the increase in concentration (bioaccessibility index >100%) observed for quercetin tri-hexoside and acylated quercetin glycosides may be attributed to deglycosylation reactions involving unidentified glycosylated derivatives of quercetin. In addition, isorhamnetin-4′-O-hexoside, isorhamnetin-3-O-hexoside, and isorhamnetin-3-O-hexoside-4′-O-hexoside also exhibited bioaccessibility indices significantly exceeding 100% (*p* < 0.05), further suggesting the hydrolysis of isorhamnetin di-hexosides and more complex glycosylated derivatives that were not identified in the present study (Table 1).

After in vitro intestinal digestion, a significant decrease (*p* < 0.05) in the amount of bioaccessible total flavonols was detected, resulting in a bioaccessible index of 79.0% (Table 1 and Figure 3).

**Figure 4:**
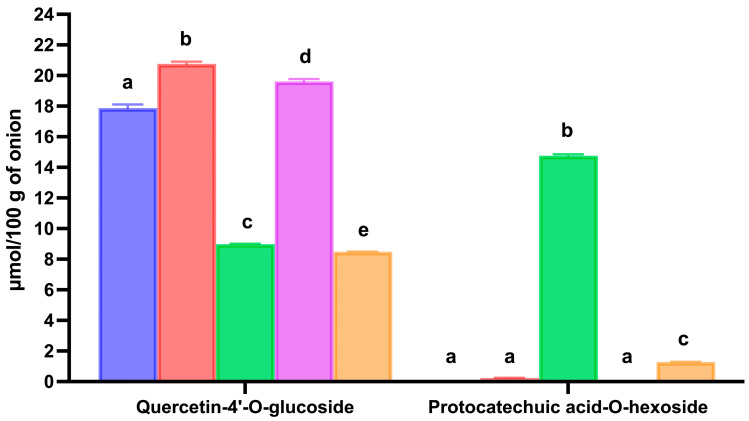
Effect of in vitro gastrointestinal digestion on the changes in quercetin-4‘-O-glucoside and protocatechuic acid-O-hexoside in red-skinned onion and red-skinned onion phenolic compounds extract. Blue bars: onion phenolic compounds extract; red bars: gastric digested onion; green bars: intestinal digested onion; purple bars: gastric digested onion extract; orange bars: intestinal digested onion extract. Error bars, mean ± S.D. Different letters within the same data group indicated statistically significantly different (*p* < 0.05) values. Significant differences were determined by ANOVA (Univariate analysis of variance) followed by Tukey’s post hoc test.

The decrease in total flavonols concentration observed from the gastric to the intestinal phase of digestion amounted to 14.05 μmol/100 g of onion and was largely attributable to a reduction in quercetin-4′-O-glucoside. Specifically, the concentration of quercetin-4′-O-glucoside declined by 11.77 μmol/100 g of onion, accounting for 83.8% of the total flavonols loss following intestinal digestion (Table 1 and Figure 4). Previous studies suggested that quercetin-mono-hexosides may undergo oxidative degradation in the alkaline intestinal milieu, leading to the production of the corresponding protocatechuic acid-hexosides [42,52,53]. As reported in Figure 4, during in vitro gastrointestinal digestion, the concentration of protocatechuic acid-O-hexoside increased from 0.06 ± 0.00 μmol/100 g of onion in the extract to 0.24 ± 0.01 μmol/100 g of onion after gastric digestion and further significantly increased (*p* < 0.05) to 14.76 ± 0.09 μmol/100 g of onion at the end of the intestinal digestion. Overall, these results indicated that the observed reduction in quercetin-mono-hexosides during intestinal digestion is largely attributable to oxidative degradation pathways resulting in the formation of protocatechuic acid-O-hexoside.

Regarding the other two most important flavonols detected in red-skinned onion, both quercetin-3-O-glucoside-4′-O-glucoside and isorhamnetin-4′-O-hexoside exhibited high bioaccessibility indices, near or above 100% (Table 1).

When red-skinned onion phenolic compounds extract was subjected to in vitro gastro-intestinal digestion, a bioaccessibility index of 103.4% was observed after the gastric phase of digestion, suggesting that onion flavonols were stable under the acidic condition of the gastric medium, independently of the presence of the food matrix (Table 1 and Figure 3). Looking at the individual compounds, a decrease of about 2 μmol/100 g of onion was seen for quercetin-3-O-glucoside-4′-O-glucoside after gastric digestion, concurrently with an increase in the same magnitude of the amount of quercetin-4′-O-glucoside. These results were consistent with the acidic hydrolysis of the glucose moiety at the 3-O- position observed during in vitro gastric digestion of red-skinned onion (Table 1).

However, after the intestinal phase of digestion, a significantly lower amount (*p* < 0.05) of total flavonols was recorded in the digested red-skinned onion phenolic compounds extract compared to the digested red-skinned onion (Table 1 and Figure 3). The calculated bioaccessibility index was 57.5%, which was lower by more than 27% compared with that observed after digestion of red-skinned onion (Table 1 and Figure 3). These results indicated that the presence of food matrix may partially protect red-skinned onion flavonols from degradation. Both quercetin-3-O-glucoside-4′-O-glucoside and quercetin-4′-O-glucoside were degraded during intestinal digestion of red-skinned onion phenolic compounds extract, exhibiting a bioaccessibility index of 60.7% and 47.5%, respectively (Table 1). As reported above for red-skinned onion digestion, quercetin derivatives may undergo oxidative degradation during in vitro intestinal digestion. However, as reported in Figure 4, in the case of the digestion of red-skinned onion phenolic compounds extract, only a low amount of protocatechuic acid-O-hexoside (1.29 ± 0.01 μmol/100 g of onion) was detected at the end of the intestinal digestion. These data suggested that during in vitro intestinal digestion of red-skinned onion phenolic compounds extract, quercetin-hexosides followed a different oxidation pathway, or that, in the absence of the food matrix, the oxidative processes were more advanced, leading to the formation of unidentified oxidation products.

A possible explanation for the protective effect of the onion food matrix on quercetin-derivatives degradation could be related to the presence in the food matrix of fiber, which can interact via non-covalent interactions with onion quercetins at the alkaline pH of the intestinal fluid [27,54,55]. In this way, fiber-bound quercetin derivatives may be released gradually during intestinal digestion, slowing down oxidative processes and enhancing the stability of flavonols. Another possibility is that the onion food matrix contained antioxidant compounds other than flavonols (such as sulfur-containing compounds), which were not present in the red-skinned onion phenolic compounds extract and may have protected flavonols from oxidation in the intestinal medium [56].

### 3.4. Identification and In Vitro Bioaccessibility of Individual Flavan-3-Ols in Dark Chocolate and Dark Chocolate Phenolic Compounds Extract

A total of 24 flavan-3-ols were identified in the phenolic compounds extract of dark chocolate (Table 2). The total amount of flavan-3-ols identified by mass spectrometry was 245.53 ± 8.56 μmol/100 g of dark chocolate (Table 2). The compound found at the highest concentration was procyanidin B1, which accounted for 35.5% of total flavan-3-ols, followed by epicatechin, which represented 20.3% of total flavan-3-ols (Table 2). In addition to epicatechin, its isomer catechin was also detected at an appreciable but 3.4-times lower amount. Three additional isomers of procyanidin-type B dimers (including procyanidin B2) were identified in the dark chocolate phenolic compounds extract, such that the total amount of dimeric procyanidin-type B was 110.86 ± 2.75 μmol/100 g of dark chocolate. Furthermore, four isomers of procyanidin-type B trimers (total amount of 30.11 ± 0.57 μmol/100 g of dark chocolate), three isomers of procyanidin-type B tetramers (total amount of 25.59 ± 2.70 μmol/100 g of dark chocolate), and one isomer of procyanidin-type B pentamers were also ascertained (Table 2). The phenolic profile of dark chocolate was in line with previously reported data [34,57].

**Figure 5:**
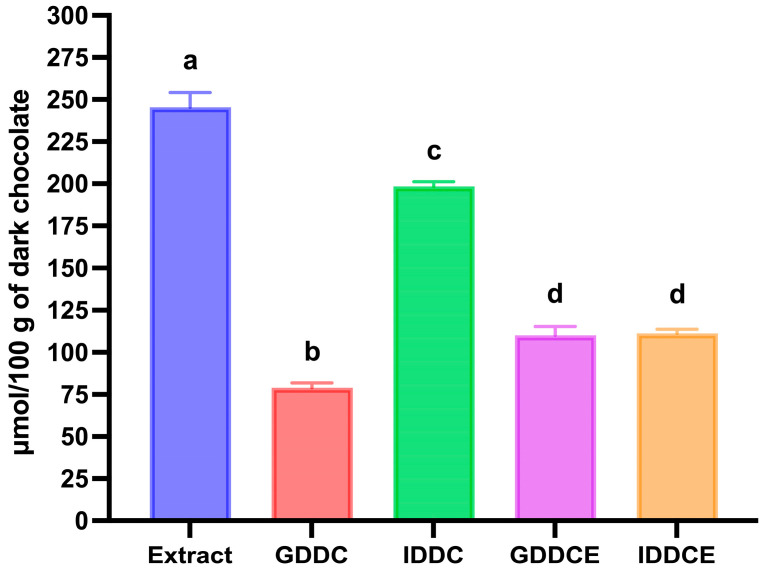
Effect of in vitro gastrointestinal digestion on the changes in total phenolic compounds determined by mass spectrometry in dark chocolate and dark chocolate phenolic compounds extract. Extract: dark chocolate phenolic compounds extract; GDDC: gastric digested dark chocolate; IDDC: intestinal digested dark chocolate; GDDCE: gastric digested dark chocolate extract; IDDCE: intestinal digested dark chocolate extract. Error bars, mean ± S.D. Different letters indicated statistically significant differences (*p* < 0.05). Significant differences were determined by ANOVA (Univariate analysis of variance) followed by Tukey’s post hoc test.

**Figure 6:**
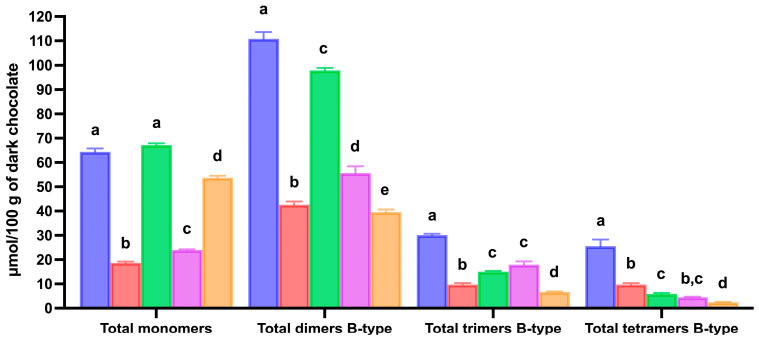
Effect of in vitro gastrointestinal digestion on the changes in total flavan-3-ols monomers, total dimeric B-type procyanidins, total trimeric B-type procyanidins, and total tetrameric B-type procyanidins in dark chocolate and dark chocolate phenolic compounds extract. Blue bars: dark chocolate phenolic compounds extract; red bars: gastric digested dark chocolate; green bars: intestinal digested dark chocolate; purple bars: gastric digested dark chocolate extract; orange bars: intestinal digested dark chocolate extract. Error bars, mean ± S.D. Different letters within the same data group indicated statistically significantly different (*p* < 0.05) values. Significant differences were determined by ANOVA (Univariate analysis of variance) followed by Tukey’s post hoc test.

After in vitro gastric digestion of dark chocolate, only 32.1% of total flavan-3-ols were released from the food matrix, whereas their bioaccessibility increased to 80.8% at the end of the intestinal digestion (Table 2 and Figure 5). At this stage, all the major flavan-3-ols exhibited bioaccessibility indices near or equal to 100%. Furthermore, intestinal digestion resulted in a progressive decrease in the bioaccessibility index of flavan-3-ols with increasing degree of polymerization, from 104.3% for total B-type monomers to 88.3% for total B-type dimers, 50.0% for total B-type trimers, and 23.3% for total B-type tetramers, indicating reduced release or stability of higher oligomers (Table 2 and Figure 6).

Regarding the digestion of the dark chocolate phenolic compounds extract, a strong and significant decrease (*p* < 0.05) in the concentration of total flavan-3-ols was detected after gastric digestion, resulting in a bioaccessibility index of 44.7% (Table 2 and Figure 5). Considering the individual compounds, the oligomeric procyanidins concentration declined significantly (*p* < 0.05) after gastric digestion. Procyanidin B1, which was present at the highest amount in the extract, displayed a relatively low bioaccessibility index of 47.8% following gastric digestion, indicating stability under gastric conditions (Table 2). A similar decrease was also observed for the other B-type dimeric procyanidins (Table 2 and Figure 6). Furthermore, the lowest bioaccessibility index was found for total B-type tetrameric procyanidins with a recovery after gastric digestion of 17.8% (Table 2 and Figure 6). Previous in vitro studies suggested that procyanidins may undergo hydrolysis under the acidic conditions of the stomach [58,59]. In particular, acid hydrolysis has been demonstrated during gastric digestion of oligomeric procyanidins with a degree of polymerization between three and six [58,59]. In addition, procyanidins may precipitate in the acidic gastric milieu due to interactions with pepsin, which could account for the observed decrease in their concentrations following gastric digestion [60,61]. Therefore, the low bioaccessibility index of dark chocolate oligomeric procyanidins after gastric digestion may be a consequence of both their hydrolysis, which occurred under acidic conditions, and their interaction with pepsin, which caused the formation of protein–procyanidin complexes that precipitated during gastric digestion.

In contrast, procyanidin A-type dimers were more stable during gastric digestion, with bioaccessibility indices for the different isomers near 100%. This could be due to the presence of an additional C2-O-C7 bond that made A-type procyanidins less susceptible to hydrolysis under the acidic gastric conditions [62].

In addition, monomeric flavan-3-ols concentration severely decreased (*p* < 0.05) after gastric digestion of dark chocolate phenolic compounds extract, resulting in a bioaccessibility index of 47.4% and 34.2% for catechin and epicatechin, respectively (Table 2 and Figure 6). This decline may be due to the binding of monomeric flavan-3-ols to pepsin under the acidic conditions of the stomach [63,64].

After intestinal digestion, the total amount of flavan-3-ols determined by mass spectrometry did not change significantly (*p* > 0.05) compared to that established after gastric digestion (Table 2 and Figure 5). However, some variations in the amounts of individual flavan-3-ols occurred. The concentration of oligomeric procyanidins decreased further after intestinal digestion (Table 2 and Figure 6). The bioaccessibility index of procyanidin B1 was 34.6% after intestinal digestion, whereas that of total B-type dimers was 35.7%. Furthermore, a strong decline in total B-type trimers and total B-type tetramers was observed after intestinal digestion, with bioaccessibility values of 22.3% and 9.7%, respectively. Previous studies found that procyanidins were degraded during intestinal digestion of chokeberry, elderberry, Brazilian açaí seeds, hawthorn, or pure procyanidin compounds [25,65,66,67]. Moreover, it has been shown that procyanidins undergo oxidative degradation under alkaline conditions, such as those found in intestinal juice [68].

Likewise, A-type dimeric procyanidins, which were stable after gastric digestion, were completely degraded at the end of the intestinal phase of digestion.

A different behavior was observed in the case of monomeric flavan-3-ols (Table 2 and Figure 6). Indeed, the concentrations of both catechin and epicatechin significantly increased (*p* < 0.05) after intestinal digestion compared with the gastric phase, reaching values close to those in the extract, with bioaccessibility indexes of 86.7% and 82.5%, respectively (Table 2 and Figure 6). This increase may be due to the release of monomers from protein–catechin complexes formed during in vitro gastric digestion due to pH changes, hydrolysis by intestinal proteases, or to the release of monomers from larger flavan-3-ols, as previously suggested [65]. Alternatively, the increased concentrations of epicatechin and catechin observed after intestinal digestion may result from their release from melanoidins. Peña-Correa and co-workers demonstrated that monomeric flavan-3-ols bound to cocoa melanoidins were readily released during in vitro digestion of high molecular-weight cocoa melanoidins [45].

Finally, a bioaccessibility index exceeding 100% was observed for (epi)catechin-mono-hexosides after intestinal digestion, likely resulting from the deglycosylation of more complex (epi)catechin hexosides not identified in the present study (Table 2).

A comparison between the data obtained after in vitro digestion of dark chocolate and its phenolic compounds extract clearly demonstrated that the food matrix had a protective effect on flavan-3-ols degradation. At the end of the intestinal digestion, the bioaccessibility of total flavan-3-ols was 80.8% in dark chocolate compared with 45.3% of its phenolic compounds extract, with all major compounds, including the monomers and the oligomeric procyanidins, exhibiting high bioaccessibility.

This protective effect of the dark chocolate matrix may be attributed to several factors. First, flavan-3-ols were gradually released from dark chocolate during digestion, with most released during the intestinal phase, thereby partially preventing gastric degradation of flavan-3-ols as observed in the phenolic compounds extract. Second, dark chocolate may contain antioxidant compounds other than flavan-3-ols (such as Maillard reaction products) that were not present in the phenolic compounds extract and that may enhance the oxidative stability of flavan-3-ols during in vitro gastrointestinal digestion [69]. Finally, the presence of fats in dark chocolate (which were removed from the phenolic compounds extract) may have protected flavan-3-ols from degradation. A protective effect of cocoa fats on flavan-3-ols during in vitro gastrointestinal digestion has been previously reported [26,70]. This protective effect may be a consequence of the interaction between lipids and flavan-3-ols, or of better micellization that improved flavan-3-ols’ stability during digestion [26,27,70].

## 4. Conclusions

Data reported in this study clearly demonstrated a protective effect of dark chocolate and red-skinned onion food matrices on the degradation of phenolic compounds during in vitro gastrointestinal digestion. Red-skinned onion flavonols and dark chocolate flavan-3-ols were more readily degraded during in vitro digestion of the extract than during digestion of the whole food. Currently, many food supplements and herbal preparations, available as capsules or liquids, contain phenolic compounds extracted from specific foods. The absence of the food matrix could alter the bioaccessibility, and consequently the bioavailability and biological activity of these compounds, potentially affecting the efficacy of these preparations. It is important to recognize that in vitro studies do not fully replicate physiological conditions. While they provide valuable insight into the mechanisms occurring in the gastrointestinal tract, they cannot fully account for the complex digestion and absorption processes in living organisms. Therefore, findings from in vitro experiments should be corroborated by further in vivo studies. However, these results underscore the importance of consuming phenolic compounds directly from vegetable-based foods, where the food matrix protects these molecules from gastrointestinal degradation, thereby maximizing their potential health-promoting and preventive effects.

## Figures and Tables

**Table 1:** Amount of flavonols in red-skinned onion phenolic extract, red-skinned onion after gastric digestion (GDO), red-skinned onion after intestinal digestion (IDO), red-skinned onion phenolic extract after gastric digestion (GDOE), and red-skinned onion phenolic extract after intestinal digestion (IDOE). Results are expressed in μmol/100 g of onion. Bioaccessibility index (BI) is the percentage ratio between the concentration after in vitro gastric or intestinal digestion and the concentration in the red-skinned onion phenolic extract. n.d. means not detected compound. Different letters indicated statistically significant differences (*p* < 0.05).

Compound	Red-Skinned Onion Flavonols								
	Extract	GDO	BI (%)	IDO	BI (%)	GDOE	BI (%)	IDOE	BI (%)
Morin	0.38 ± 0.01 ^a^	0.84 ± 0.03 ^b^	218.1	0.40 ± 0.00 ^a^	102.8	0.43± 0.01 ^a^	111.8	0.36 ± 0.00 ^a^	92.4
Quercetin	0.44 ± 0.01 ^a^	0.95 ± 0.02 ^b^	215.4	0.01 ± 0.00 ^c^	2.2	0.43 ± 0.00 ^a^	97.3	0.01 ± 0.00 ^c^	3.3
Isorhamnetin	0.11 ± 0.00	n.d.	0.0	n.d.	0.0	n.d.	0.0	n.d.	0.0
Quercetin-3-O-glucoside	0.49 ± 0.00 ^a^	0.84 ± 0.00 ^b^	170.4	0.46 ± 0.00 ^a^	92.6	0.77 ± 0.00 ^b^	155.4	0.78 ± 0.01 ^b^	159.1
Quercetin-4′-O-glucoside	17.87 ± 0.24 ^a^	20.76 ± 0.15 ^b^	116.2	8.99 ± 0.02 ^c^	50.3	19.61 ± 0.16 ^d^	109.8	8.49 ± 0.01 ^e^	47.5
Isorhamnetin-3-O-hexoside	0.09 ± 0.00 ^a^	0.14 ± 0.00 ^a^	159.2	0.20 ± 0.00 ^b^	229.7	0.13 ± 0.00 ^a^	147.2	0.12 ± 0.00 ^a^	133.7
Isorhamnetin-4′-O-hexoside	2.53 ± 0.01 ^a^	4.35 ± 0.15 ^b^	172.1	3.40 ± 0.04 ^c^	134.5	3.25 ± 0.06 ^d^	128.5	2.49 ± 0.04 ^a^	98.5
Kaempferol-O-acetylhexoside	1.17 ± 0.02 ^a^	1.50 ± 0.01 ^b^	128.4	0.01 ± 0.01 ^c^	0.8	1.42 ± 0.02 ^d^	122.1	0.17 ± 0.03 ^e^	14.7
Quercetin-O-acetylhexoside isomer 1	n.d.	0.01 ± 0.00 ^a^	173.2	n.d.	0.0	0.01 ± 0.00 ^a^	163.4	0.03 ± 0.00 ^a^	936.6
Quercetin-O-acetylhexoside isomer 2	0.04 ± 0.00 ^a^	0.06 ± 0.00 ^a^	138.0	n.d.	0.0	0.05 ± 0.00 ^a^	124.3	0.03 ± 0.00 ^a^	79.4
Kaempferol-O-hexoside-hexoside	0.40 ± 0.00 ^a^	0.44 ± 0.00 ^a^	108.9	n.d.	0.0	0.54 ± 0.00 ^b^	135.1	n.d.	0.0
Quercetin-3-O-glucoside-4′-O-glucoside	19.50 ± 1.10 ^a^	16.86 ± 0.28 ^b^	86.5	19.29 ± 0.09 ^a^	98.9	17.57 ± 0.02 ^c^	90.1	11.83 ± 0.00 ^d^	60.7
Isorhamnetin-3-O-hexoside-4′-O-hexoside	0.86 ± 0.02 ^a^	1.87 ± 0.01 ^b^	218.9	1.80 ± 0.02 ^b^	210.4	1.17 ± 0.01 ^c^	136.8	0.86 ± 0.00 ^a^	100.2
Quercetin-O-hexoside-O-malonylhexoside isomer 1	0.03 ± 0.00 ^a^	0.06 ± 0.00 ^a^	203.6	0.06 ± 0.00 ^a^	195.8	0.01 ± 0.00 ^a^	22.9	0.03 ± 0.00 ^a^	118.0
Quercetin-O-hexoside-O-malonylhexoside isomer 2	0.04 ± 0.00 ^a^	0.06 ± 0.01 ^a^	155.3	0.06 ± 0.00 ^a^	169.7	0.05 ± 0.00 ^a^	132.4	0.03 ± 0.00 ^a^	96.6
Quercetin-tri-O-hexoside	0.09 ± 0.00 ^a^	0.12 ± 0.00 ^a^	138.6	0.14 ± 0.00 ^a^	153.9	0.11 ± 0.00 ^a^	125.1	0.10 ± 0.00 ^a^	111.0
Total flavonols	44.03 ± 1.41 ^a^	48.85 ± 0.67 ^b^	111.00	34.80 ± 0.19 ^c^	79.00	45.55 ± 0.28 ^a^	103.4	25.34 ± 0.08 ^d^	57.5

**Table 2:** Amount of flavan-3-ols in dark chocolate phenolic extract, dark chocolate after gastric digestion (GDDC), dark chocolate after intestinal digestion (IDDC), dark chocolate phenolic extract after gastric digestion (GDDCE), and dark chocolate phenolic extract after intestinal digestion (IDDCE). Results are expressed in μmol/100 g of dark chocolate. Bioaccessibility index (BI) is the percentage ratio between the concentration after in vitro gastric or intestinal digestion and the concentration in the dark chocolate phenolic extract. n.d. means not detected compound. Different letters indicated statistically significant differences (*p* < 0.05).

Compound	Dark Chocolate Flavan-3-Ols								
	Extract	GDDC	BI (%)	IDDC	BI (%)	GDDCE	BI (%)	IDDCE	BI (%)
Catechin	14.69 ± 0.24 ^a^	4.85 ± 0.03 ^b^	33.0	15.65 ± 0.10 ^c^	106.5	6.97 ± 0.09 ^d^	47.4	12.74 ± 0.22 ^e^	86.7
Epicatechin	49.73 ± 1.09 ^a^	13.83 ± 0.50 ^b^	27.8	51.55 ± 0.59 ^a^	103.6	17.01 ± 0.19 ^c^	34.2	41.02 ± 0.54 ^d^	82.5
Gallocatechin	0.08 ± 0.00 ^a^	0.06 ± 0.00 ^a^	76.6	0.08 ± 0.01 ^a^	104.9	0.10 ± 0.00 ^a^	127.2	0.04 ± 0.00 ^a^	58.6
(Epi)catechin-O-hexoside isomer 1	0.68 ± 0.00 ^a^	0.26 ± 0.02 ^b^	37.6	0.72 ± 0.00 ^a^	104.9	0.41 ± 0.14 ^c^	60.7	0.67 ± 0.01 ^a^	98.3
(Epi)catechin-O-hexoside isomer 2	1.00 ± 0.02 ^a^	0.28 ± 0.03 ^b^	27.8	1.18 ± 0.39 ^a^	117.6	0.30 ± 0.01 ^b^	30.0	0.71 ± 0.02 ^c^	70.7
(Epi)catechin-O-hexoside isomer 3	1.27 ± 0.02 ^a^	0.84 ± 0.00 ^b^	65.9	1.99 ± 0.01 ^c^	156.3	0.77 ± 0.11 ^b^	60.3	1.52 ± 0.04 ^d^	120.0
(Epi)catechin-C-hexoside isomer 1	1.57 ± 0.04 ^a^	1.09 ± 0.02 ^b^	69.2	2.91 ± 0.09 ^c^	185.1	1.08 ± 0.01 ^b^	68.5	2.14 ± 0.04 ^d^	136.0
(Epi)catechin-C-hexoside isomer 2	0.28 ± 0.02 ^a^	1.59 ± 0.05 ^b^	569.2	3.45 ± 0.05 ^c^	1231.2	1.62 ± 0.02 ^b^	578.5	2.52 ± 0.06 ^d^	898.6
(Epi)catechin-C-hexoside isomer 3	0.21 ± 0.00 ^a^	0.22 ± 0.01 ^a^	102.5	0.39 ± 0.00 ^b^	182.0	0.23 ± 0.01 ^a^	108.9	0.32 ± 0.02 ^b^	148.7
Procyanidin-type A dimer isomer 1	0.59 ± 0.04 ^a^	0.10 ± 0.02 ^b^	17.2	n.d.	0.0	0.48 ± 0.01 ^c^	82.5	n.d.	0.0
Procyanidin-type A dimer isomer 2	0.51 ± 0.01 ^a^	0.12 ± 0.01 ^b^	22.7	n.d.	0.0	0.48 ± 0.02 ^a^	93.9	n.d.	0.0
Procyanidin-type A dimer isomer 3	0.35 ± 0.02 ^a^	0.09 ± 0.01 ^b^	26.7	n.d.	0.0	0.49 ± 0.01 ^c^	138.1	n.d.	0.0
Procyanidin-type B tetramer isomer 1	1.99 ± 0.44 ^a^	0.21 ± 0.08 ^b^	10.6	0.56 ± 0.07 ^c^	28.1	0.59 ± 0.11 ^c^	29.5	0.43 ± 0.02 ^c^	21.4
Procyanidin-type B tetramer isomer 2	8.90 ± 1.37 ^a^	1.15 ± 0.14 ^b^	12.9	2.43 ± 0.13 ^c^	27.4	1.79 ± 0.04 ^d^	20.1	0.72 ± 0.09 ^e^	8.1
Procyanidin-type B tetramer isomer 3	14.70 ± 0.89 ^a^	1.21 ± 0.01 ^b^	8.2	2.96 ± 0.15 ^c^	20.1	2.17 ± 0.04 ^d^	14.8	1.32 ± 0.06 ^b^	9.0
Procyanidin B2	7.41 ± 0.51 ^a^	2.82 ± 0.28 ^b^	38.1	9.12 ± 0.26 ^c^	123.1	3.64 ± 0.55 ^b^	49.1	6.47 ± 0.22 ^d^	87.2
Procyanidin-type B dimer isomer 1	4.70 ± 0.19 ^a^	1.86 ± 0.10 ^b^	39.5	4.57 ± 0.13 ^a^	97.3	1.94 ± 0.79 ^b^	41.3	1.75 ± 0.13 ^b^	37.2
Procyanidin B1	87.08 ± 1.90 ^a^	33.82 ± 0.88 ^b^	38.8	79.97 ± 0.49 ^c^	91.8	41.59 ± 1.24 ^d^	47.8	30.10 ± 0.67 ^e^	34.6
Procyanidin-type B dimer isomer 2	11.66 ± 0.16 ^a^	4.17 ± 0.06 ^b^	35.7	4.27 ± 0.10 ^b^	36.6	8.48 ± 0.23 ^c^	72.7	1.24 ± 0.04 ^d^	10.6
Procyanidin-type B pentamer	8.01 ± 1.04 ^a^	0.60 ± 0.03 ^b^	7.5	1.57 ± 0.04 ^c^	19.6	1.80 ± 0.22 ^c^	22.4	0.80 ± 0.04 ^d^	10.0
Procyanidin-type B trimer isomer 1	1.67 ± 0.06 ^a^	0.66 ± 0.23 ^b^	39.2	1.29 ± 0.03 ^c^	77.1	1.84 ± 0.73 ^a,c^	109.8	1.09 ± 0.05 ^c^	65.2
Procyanidin-type B trimer isomer 2	5.75 ± 0.13 ^a^	1.99 ± 0.15 ^b^	34.6	2.03 ± 0.07 ^b^	35.3	3.62 ± 0.37 ^c^	62.9	0.51 ± 0.03 ^d^	8.9
Procyanidin-type B trimer isomer 3	18.41 ± 0.27 ^a^	6.59 ± 0.14 ^b^	35.8	10.37 ± 0.13 ^c^	56.3	10.33 ± 0.18 ^c^	56.1	4.62 ± 0.10 ^d^	25.1
Procyanidin-type B trimer isomer 4	4.27 ± 0.11 ^a^	0.55 ± 0.04 ^b^	13.0	1.36 ± 0.06 ^c^	31.9	2.16 ± 0.07 ^d^	50.6	0.50 ± 0.02 ^b^	11.7
Total flavan-3-ols	245.53 ± 8.56 ^a^	78.94 ± 2.83 ^b^	32.1	198.41 ± 2.89 ^c^	80.80	109.87 ± 5.19 ^d^	44.70	111.22 ± 2.43 ^d^	45.30

## Data Availability

The original contributions presented in the study are included in the article/Supplementary Material; further inquiries can be directed to the corresponding author.

## Supplementary Materials

The following supporting information can be downloaded at https://www.mdpi.com/article/10.3390/biology15010088/s1, Table S1: Mass spectrometry data of identified flavonols and flavan-3-ols.
